# Management of Invasive Fungal Infections in Adult Patients with Hematological Malignancies in Greece during the Financial Crisis: Challenges and Recommendations

**DOI:** 10.3390/jof4030094

**Published:** 2018-08-09

**Authors:** Nikolaos V. Sipsas, Maria N. Pagoni, Diamantis P. Kofteridis, Joseph Meletiadis, Georgia Vrioni, Maria Papaioannou, Anastasia Antoniadou, George Petrikkos, George Samonis

**Affiliations:** 1 Infectious Diseases Unit, Laiko General Hospital and Medical School, National and Kapodistrian University of Athens, 11527 Athens, Greece; 2 Haematology-Lymphomas Department and BMT Unit, Evangelismos Hospital, 10676 Athens, Greece; marianpagoni@yahoo.com; 3 Department of Internal Medicine, Infectious Diseases Unit, University of Crete Medical School, Heraklion, 71500 Crete, Greece; kofterid@med.uoc.gr (D.P.K.); samonis@med.uoc.gr (G.S.); 4 Clinical Microbiology Laboratory, Attikon University Hospital, National and Kapodistrian University of Athens, 12462 Athens, Greece; jmeletiadis@med.uoa.gr; 5 Department of Microbiology, Medical School, National and Kapodistrian University of Athens, 11527 Athens, Greece; gvrioni@med.uoa.gr; 6 Hematology Unit, First Department of Internal Medicine, AHEPA University Hospital, Aristotle University of Thessaloniki, 54621 Thessaloniki, Greece; marygpap@gmail.com; 7 4th Department of Internal Medicine, University General Hospital Attikon, National and Kapodistrian University of Athens, 12462 Athens, Greece; ananto@med.uoa.gr (A.A.); petrikos@hol.gr (G.P.)

**Keywords:** invasive fungal infections, hematological malignancy, financial crisis, Greece

## Abstract

There are concerns that the financial crisis in Greece negatively affected the management of invasive fungal infections (IFIs) among patients with hematological malignancies (HM). A working group (WG) was formed to explore the situation and make recommendations. A questionnaire was created and distributed to physicians caring for patients with HM, to gather information in a standardized manner on prescribing physicians, patient characteristics, availability of diagnostics, antifungal treatment practices and the conditions and particularities of Greek hospitals. A total of 141 physicians from 36 hematology units and laboratories located in 26 Greek hospitals participated. Regarding hospitalization conditions, only 56% reported that their patients were treated in isolated single or double bed rooms, 22% reported availability of HEPA filters, 47% reported construction works in progress, and an alarming 18% reported the presence of birds on open windows. Regarding diagnosis, only 31% reported availability of biomarkers for diagnosis of IFIs, 76% reported that CT scans were performed in a timely fashion, 42% reported prompt availability of broncho-alveolar lavage, and only 6% availability of therapeutic drug monitoring. Of concern, 26% of the responders reported non-availability of some antifungals. In conclusion, significant challenges exist for the optimal management of IFIs in patients with HM in Greece.

## 1. Introduction

Invasive fungal infections (IFIs) are associated with considerable morbidity and mortality among patients with hematological malignancies (HM) [1]. The economic burden of IFI-related hospitalizations is also substantial, as they are associated with prolonged hospital stay and increased hospital costs [2]. The standards of care for patients with HM include, among others, availability of laminar airflow isolation rooms with HEPA filters for prevention of IFIs, especially in the hematopoietic stem cell transplantation (HSCT) units, biomarkers for early diagnosis of IFIs, specialized and well-equipped microbiology laboratory with rapid turnaround times, with 24/7 availability of imaging facilities, as well as therapeutic drug monitoring (TDM), and access to all antifungal agents [1].

The currently evolving financial crisis, that hit Greece in late 2008, resulted in drastic curtailing of government spending, which significantly affected public hospitals that cope with understaffing, deficits, and shortage of drugs and basic medical supplies [3]. The significant reduction in funding of health and healthcare raised concerns that the management of IFIs among patients with HM would be negatively affected, due to under-diagnosis, delayed diagnosis, limited access to treatment or inadequate care. The situation might be further complicated by possible gaps in the knowledge of Greek physicians on the optimal management of IFIs as well as in their relevant training. European studies captured heterogeneity in experience and education among European physicians [4,5]. 

A working group (WG) has been instituted to assess the situation and identify the problems regarding the management of IFIs among patients with HM treated in Greek hospitals during the financial crisis. The aims were to delineate how the existing situation affects the everyday clinical practice of the treating physicians and develop a set of recommendations based on the Greek reality, trying to keep in accordance to what is recommended by the published international guidelines.

## 2. Materials and Methods

The present study has been developed under the auspices of the Hellenic Society of Medical Mycology, and the Hellenic Society of Hematology, by a WG consisting of two hematologists, five ID physicians, and two microbiologists working at tertiary care Greek hospitals with experience and active clinical practice in the management of IFIs in patients with HM. Initially, a questionnaire was created to gather information in a standardized manner on prescribing physicians, patient characteristics, availability of diagnostics, antifungal treatment practices, and the conditions of Greek hospitals pertaining to the optimal care of these high-risk patients. The questionnaire requested information on the type of hospital, presence of special units (infectious disease unit, hematology unit, HSCT unit, radiology and microbiology departments) and number of admitted patients with HM, per year. The form contained standards of care questions for the four main areas, namely, clinical medicine, microbiology, histopathology, and radiology, requiring a yes or no answer. An option for free text feedback was also provided. 

In brief, the diagnostic section required information on microscopy, culture and fungal speciation from several different specimens, (blood, urine, intra-vascular line tips, bronchoscopy and bronchoalveolar lavage (BAL) and other specimens), as well as information on the use of specific tests for the detection of fungal infection including antigen/antibody detection (galactomannan (GM), β-d-glucan (BG), mannan (MN), anti-mannan (anti-MN)) and molecular assays. The radiology section required information on the availability of computer tomography (CT) scans and other imaging in high-risk patient groups and speed of scan review. The clinical information included the facilities (private rooms, ongoing construction works, level of cleanliness, effective infection control), availability of necessary microbiological tests, timely imaging, access to antifungal agents, availability of TDM, use of prophylaxis and in what patient populations, therapeutic strategy (pre-emptive vs empiric) for the patient with neutropenia and fever, and the availability of continuous medical education.

The questionnaires were distributed by the members of the committee and their collaborators to physicians prescribing antifungal agents (ID physicians and hematologists), and microbiologists, in 26 hospitals located in 9 Greek cities. These 26 hospitals, are the only hospitals, among a total of 136 Greek hospitals, who serve patients with hematological malignancies, therefore, they cover 100% of such Greek patients. Specific questionnaires on the availability of diagnostic methods were also sent to the heads of 17 hospital laboratories. The participating hospitals had one to four different active hematology units. Whilst the questionnaire was not formally validated prior to the study, its design was discussed with expert statisticians and then reviewed in the initial stages of the project. No questionnaire modifications were deemed necessary and the study was continued as initially planned.

The results of the audit have been analyzed and the WG defined the main challenges in the management of IFIs among patients with HM in the era of austerity measures, and in subsequent consecutive meetings adjusted the existing recommendations to the Greek reality. The final document has been prepared in consensus, and all members approved the final draft.

## 3. Results

The main results of our survey are shown in Table 1, Table 2, Table 3 and Table 4. There were no recorded instances of refusal by a physician to participate in our study, probably because in most cases the questionnaires have been handed to physicians personally by members of the WG. A total of 140 physicians and one PhD biologist from 36 hematology units located in 26 Greek hospitals participated; 114 (81%) of them were hematologists, 17 (12%) ID physicians, and the rest internists (*n* = 1) and microbiologists (*n* = 9); 34% of the physicians were experienced hematology fellows. Most participating (52%) physicians were treating patients with acute leukemia, while only 11% were treating patients with allogeneic hematopoietic stem cell transplantation. 

### 3.1. Conditions of Hospitalization

Our survey, showed poor hospitalization conditions. More specifically, only 56% of the physicians reported that their high-risk patients with acute leukemia were treated in isolated, single or double bed rooms. In 44% of the cases, patients with HM were treated in common hematology rooms with 3–6 beds (26%), in the internal medicine wards (13%), or even in hospital corridors lying on stretchers (5%). Availability of HEPA filters was reported by 22% of responders. Construction work in progress, within the hospitals, was reported by 47% of participants, but appropriate infection control measures were in place in only 51% of these cases. This is in accordance with a recent study on poor hand hygiene practices in Greece [6]. Finally, an alarming 18% of the participants reported as an important problem, the presence of birds (usually pigeons) on the open windows and even in the hospital rooms. 

### 3.2. Diagnostic Capacity

A questionnaire on the available diagnostic modalities was distributed to participating clinicians. According to their response (Table 2), only 31% reported use of biomarkers for the diagnosis of IFIs, 76% reported timely performance of CT scans, while only 42% reported prompt access to bronchoscopy and BAL culture. Only 26% of physicians reported turnaround times <48 h from the time of sending the sample for the laboratory results, including biomarkers, blood cultures and species identification, histology and cytology, as well as radiology. 

To assess the diagnostic capacity, an additional questionnaire was sent to the heads of 17 microbiological laboratories of the participating hospitals. According to the provided answers the situation in diagnostics is as follows (Table 3): 33% of laboratories can identify the fungus in genus and species level in the case of *Candida* and *Aspergillus*, but only 19% in the case of other yeasts or molds. The identification of the isolated fungus is made mainly by its characteristics in culture (macro/micro) (29%), the germ tube test in the case of *Candida* (26%), as well as with automated systems (Vitek, bioMerieux, Durham, NC, USA) (29%); only 53% of the laboratories have the capacity to perform GM tests, and 13% BG. Additionally, having the capacity does not translate into routine testing in most laboratories, due to lack of funding. All laboratories do antifungal susceptibility testing in case of invasive candidiasis (half of them with commercial kits), but only 10% test for *Aspergillus* or other molds. Unfortunately, while 6% of the labs could measure plasma concentration of anti-fungal drugs, none of the participating physicians reported usage of TDM. 

### 3.3. Treatment Strategies

According to the results of our questionnaire, 95% of respondents use antifungal prophylaxis not only in high risk patients (i.e., acute myeloid leukemia (AML) patients receiving induction chemotherapy (22%)), but also in AML patients receiving consolidation therapy (18%), lymphoma (37%), multiple myeloma (9%), or any HM patient not treated under optimal environmental conditions (9%).

The questionnaire to clinicians (Table 2) revealed that only 31% of the responders reported availability of biomarkers in their clinical practice. Additionally, only 26% of the responders reported delivery of the laboratory results within 24–48 h. Regarding the CT scan, 26% of the physicians reported that it was not immediately available, while only 42% reported easy access to bronchoscopy and BAL. These clinical realities make preemptive antifungal therapy infeasible in most Greek hospitals; therefore, only 21% of the responders reported use of this approach, while 59% use the empirical strategy, i.e., initiation of antifungals in febrile, neutropenic patients not responding to broad-range antibiotics, including antibiotics active against multi-drug resistant Gram-negative pathogens, which are endemic in Greek hospitals [7,8,9,10,11,12]. The timing of initiation of empirical antifungal treatment in Greek hospitals was in accordance with International Guidelines 8, i.e., 3–5 days (52% of the responders) or 6–8 days (41%) of febrile neutropenia not responding to broad-spectrum antibiotics (Table 4).

### 3.4. Antifungal Agents

The factors affecting the choice of an antifungal agent for the initial empirical treatment by Greek physicians are shown on Table 4. Cost is the major factor (28%) affecting the choice of antifungal treatment, a factor that was non-existent in the pre-crisis era, according to the personal experience of the members of the WG. A substantial proportion of responders (26%) reported occasional non-availability of specific antifungal drugs in their hospitals due to budget constraints and/or to suboptimal logistics and organization of the pharmacy. Although the majority (66%) of Greek physicians recognized the need for determination of blood levels of certain antifungals, only 6% of the responders reported availability of such an assay, a fact not favoring the use of antifungals requiring regular blood level monitoring. 

## 4. Discussion and WG Recommendations

Our survey showed poor hospitalization conditions, as depicted on Table 1, and thus an increased risk for IFIs can be expected. Unfortunately, epidemiological data on the incidence of IFIs among patients with HM in Greece are scarce due to the lack of surveillance systems and population-based epidemiological studies [13]. In a recent multicenter study of candidemia in patients with HM, an incidence of 1.4 cases/1000 admissions was recorded. Non-*albicans* strains predominated, with *C. parapsilosis* being the leading cause of candidemia [14]. Regarding invasive aspergillosis (IA), a study showed that 11% of patients with ΗΜ, in 3 Greek centers were positive for circulating galactomannan [15]. Finally, data on mucormycosis in Greece are sparse and flawed by reporting biases. 

The WG, based on the existing data and the poor conditions of hospitalization, believes that physicians should expect or suspect a higher incidence of IFIs among patients with HM, than that reported in the literature. The WG strongly suggests the conduct of well-designed, state-funded epidemiological studies to calculate the burden of IFIs among patients with HM, locally and nationally. 

Early diagnosis is of paramount importance for the management of IFIs. As shown in Table 2 and Table 3, both clinicians and the heads of participating laboratories, report low availability of serology, and bronchoscopy, as well as slow turnaround times. The WG believes that GM and BG testing should be available as adjunct diagnostic tests in hospitals caring for patients with HM. To this end, reference labs or centers should be established. These labs, well-equipped and staffed with personnel trained in the field of mycology, should perform all the non-culture methods quickly and accurately. CT scans, BAL and all other laboratory tests should be performed in a timely fashion. 

The poor hospitalization conditions and the subsequent increased risk for IFIs justify the extremely high proportion (95%) of Greek patients with HM receiving AF prophylaxis, a fact which explains the relatively low incidence of candidemia in this population [14]. The WG believes that physicians should consider antifungal prophylaxis based not only on patients’ risk factors, but also on the existing conditions of hospitalization, and environmental factors. The WG adopts the recent ECIL recommendations [12] and suggests tabs of posaconazole (300 mg q12h × 2 → 300 mg q24h, oral) or fluconazole (400 mg q24h IV/oral) when the risk for mold infections is low. Alternatively, voriconazole (200 mg q12h oral) or micafungin (100 mg q24h IV) are recommended. The WG recommends against itraconazole as prophylaxis, based on high resistance rates reported by the recent Greek candidemia study [14,16].

Preemptive antifungal therapy is based upon the results of serial screening for aspergillosis, and therefore, upon the ability of laboratories to perform serial diagnostic tests and deliver the results in a timely fashion [17]. The Greek laboratories, as shown in Table 2 and Table 3, cannot support preemptive therapy, therefore, only 21% of physicians use this strategy; the majority (59%) use empirical AF therapy. The decision to start empirical antifungal therapy in the setting of febrile neutropenic patients with HM depends heavily on the risk of the patient for IFIs. If we consider that 47% of the responders reported active construction works in their hospitals without precautionary measures (51%), and sub-optimal hospitalization conditions (Table 1), we can assume that a substantial proportion of hospitalized Greek patients with HM are exposed to additional environmental risk factors for IFIs, a fact dictating the early institution of empirical antifungal treatment.

Taking into consideration the efficacy and safety issues associated with preemptive therapy [18,19] and the current Greek hospital reality, which does not allow adequate microbiological and radiological support, the WG considers the empirical approach as the most reasonable choice, associated with a better outcome, despite the risk of over-treating patients without IFIs. However, preemptive therapy might be considered as an alternative for clinically stable patients, in those Greek centers, where a risk-based approach is feasible by using a structured monitoring program and specific clinical rules to identify patients with IFIs in a timely manner.

After considering the abundance of environmental risk factors for IFIs in Greek hospitals, the suboptimal infection control policies, and the relative lack of appropriate barrier precautions, the WG recommends initiation of empirical antifungal treatment after 3–5 days of fever, despite administration of appropriate broad-spectrum antibiotics, and if the overall duration of neutropenia is expected to be >7 days. In clinically unstable, severely ill, high-risk patients, empirical initiation of antifungal treatment might be considered even earlier. 

Regarding the selection of AF for empirical treatment, the lack of epidemiological data on mold infections among Greek patients with HM, the widespread use of antifungal prophylaxis, the results of major international clinical studies [20,21,22], and international guidelines [12] have been considered by the WG. For patients not receiving prophylaxis, or receiving fluconazole prophylaxis, liposomal amphotericin B (3 mg/kg) and caspofungin (loading dose of 70 mg, then 50 mg/day) are recommended as the initial empirical choice, and voriconazole (loading dose of 6 mg/kg, twice per day and then 4 mg/kg) as an alternative (although not approved by any National Medicinal Authority for the indication of empirical therapy). For patients receiving prophylaxis with an azole with anti-mold activity (namely voriconazole and posaconazole), liposomal amphotericin B and caspofungin are recommended. For patients receiving prophylaxis with an echinocandin, liposomal amphotericin B and voriconazole are recommended. Micafungin can be used as an alternative to caspofungin for empirical therapy, although it is not approved for this indication [12]. 

Echinocandins should be used with caution for empirical therapy in centers with high prevalence of candidemia due to *Candida* strains with reduced susceptibility to echinocandins. Voriconazole should be used with caution in centers that do not have TDM capability. 

The same limitations that affect the empirical treatment apply to the selection of targeted treatment. According to the input of 17 laboratories, 84% of diagnosed IFIs in patients with HM were invasive *Candida* infections, namely candidemia. This suggests a reduced capacity for microbiological diagnosis of IA, probably due to lack of equipment and resources. Moreover, the non-availability of GM testing makes the diagnosis of “probable” IA, according to the EORTC criteria [23], infeasible for most participating physicians. Usually, the caring physicians rely on the clinical picture and the CT scan findings (when available) to initiate “targeted” treatment for invasive aspergillosis. As in empirical treatment, the non-availability of all antifungal drugs (26%), and of TDM (94%) affect the choice of targeted treatment. In a center where TDM was performed, large inter-(73%) and intra-(up to 85%) individual variation of voriconazole serum levels was found with a significant number of patients having sub-therapeutic (<2 mg/L) or toxic (>5 mg/L) levels (48% and 17%, respectively) [24].

Regarding candidemia, 22% of physicians reported that their laboratory does not have the capacity for species identification and an additional 34.5% reported non-availability of antifungal susceptibility testing. The existing epidemiological data suggest that non-*albicans Candida* spp, and especially *C. parapsilosis* predominate in the Greek hematology units [14]. Although 27% of the isolated strains were resistant to at least one antifungal agent [16], resistance to echinocandins was not an issue. 

The WG believes that IA should be considered in neutropenic patients with findings on the CT, even if GM testing is not available. The WG taking into account recently published international guidelines [25,26] considers voriconazole as the antifungal agent of choice, with liposomal amphotericin B being an alternative. Inadequate serum levels of voriconazole should be suspected in the absence of TDM when there is no clinical response. A higher maintenance voriconazole dose of 300 mg may provide better exposure, particularly in the absence of TDM [24,27]. In the case of prophylaxis with a mold-active antifungal agent, switching to another class of antifungals is suggested. 

For candidemia, based on the existing epidemiological data and international guidelines [25,28], the WG suggest an echinocandin or liposomal Amphotericin B as the initial empiric therapy until the susceptibility testing becomes available. In instances where species identification or susceptibility testing is not available, local epidemiology should be considered, and an antifungal agent with no resistance issues, i.e., an echinocandin or liposomal Amphotericin B should be prescribed. Isavuconazole was not available in Greece, when this document was drafted; therefore, it is not included in the recommendations. 

Data on mucormycosis and other rare molds are scarce in Greece, therefore the WG has no further recommendations to make other than to follow the international guidelines [25,29]. 

Our survey has limitations as there might be selection and reporting biases confounding the results. The WG acknowledges that there are no data from the pre-crisis era, therefore the problems identified might not be entirely a result of austerity measures, but could have existed previously to a certain point, due to poor infrastructure, lack of epidemiological data, infection control issues, and educational gaps. According to the personal experience of the members of the WG the situation in their respective hospitals worsened dramatically during the crisis. Although multi-bed rooms existed in the past, the non-availability of certain antifungals, the slow turnaround times, and the lack of serology are results of austerity. What is certain, however, is that the management of IFIs in patients with HM has not been improved during the nine years of crisis, as it would be expected if the country had continuous economic growth. Other limitations are that factors affecting the management of IFIs, such as crisis-induced stress on personnel and on public hospitals [3] were not assessed. Discrepancies among answers provided by physicians from the same hospital were not assessed as well. Yet, this was the first effort to get a picture of the management of IFIs in the era of austerity. Finally, we should keep in mind that although IFIs represent a significant threat, in Greece, the major infectious complications in patients with HM are due to multi-drug resistant gram-negative (MDRGN) pathogens, especially carbapenem-resistant enterobacteriacae, such as *Pseudomonas aeruginosa Klebsiella pneumoniae, Acinetobacter baumannii*, and *Stenotrophomonas maltophilia*. Greece is considered endemic for MDRGN pathogens, and these infections are associated with unacceptably high mortality rates among patients with HM, exceeding 50% in a recent study [30]. 

## 5. Conclusions

In conclusion, the WG believes that the management of IFIs in patients with HM is currently suboptimal in Greece. Undoubtedly, the major reduction in funding for hospitals is associated with shortage of some antifungal agents, at least for certain periods, non-availability of biomarkers as well as TDM, poor hospitalization conditions predisposing patients to higher risk of IFIs, slow laboratory turnaround times, and delays in the imaging. Lack of reliable epidemiological data is another hardship in selecting the proper treatment. Physicians try to cope by using prophylaxis even in low risk patients and by administering empirical antifungal therapy based solely on clinical and imaging criteria. Under the current situation of financial austerity, the WG suggests feasible measures such as the institution of centralized laboratories, implementation of antifungal stewardship programs, use of generics when available, and conduct of epidemiological studies. Emphasis should be given to risk stratification to avoid excessive antifungal drug expense and overexposure of low risk patients [31]. Finally, training of the prescribing physicians on the appropriate use of antifungals in patients with HM is the cornerstone of any intervention, as there are considerable knowledge gaps [4,5,6]. Our recommendations could also be applicable in other countries in financial crisis or with limited resources, considering geo-climatic and social differences that might influence epidemiology.

## Figures and Tables

**Table 1:** Prescribing physicians, facilities, and hospital conditions affecting the risk for and management of invasive fungal infections (IFIs) in Greek Hospitals.

Prescribing Physicians		%
Specialty	Hematologist	81
	Infectious Diseases	12
	Other	7
Level of experience	Specialist-attending physician	66
	Resident	34
High risk patients treated by the physician	Acute leukemia	52
	Autologous HSCT	37
	Allogeneic HCST	11
**Facilities**		
Type of rooms where HM patients are treated	Isolated rooms with HEPA filters and laminar air flow systems	22
	Single- or two-bed room in hematology unit	34
	>2 beds in hematology unit	26
	>2 beds in general internal medicine wards	13
	In the corridors of the ward	5
Construction works ongoing in hospital	Yes	47
	No	53
In case of construction works, infection control measures	Yes	51
	No	49
Particular hospital conditions affecting the risk for IFIs	Birds on the windows	18
	Slow laboratory turnaround times	18
	Poor compliance to hygiene rules	9
	Irrational spatial planning of the hospital	9
	Complete lack of isolation rooms	8
	Lack or poor function of the infection control service	8
	Inadequacy of laboratories	6

HSCT: hematopoietic stem cell transplantation, HM: hematological malignancies, IFIs: invasive fungal infections.

**Table 2:** Clinicians’ take on the diagnostics of IFIs in patients with hematological malignancies (HM).

Question	Answer	%
Microbiological methods for diagnosis of IFIs during last year	Blood cultures	37
	Serology	31
	Galactomannan	26
	β-d-glucan	5
	Histology	19
	Molecular techniques	13
Laboratory turnaround time for mycology tests	24–48 h	26
	48 h–one week	65
	>1 week	9
In case of a positive culture capacity for species identification	Yes	78
	No	22
Availability of susceptibility testing to antifungals	Yes	65.5
	No	34.5
Availability of timely bronchoscopy and BAL feasibility	Easily	42
	Difficult	34
	Impossible	24
Consideration of TDM usefulness	Yes	66
	No	34
Availability of TDM	Yes	6
	No	94
Time for a CT scan performance	Immediately	76
	Delayed	18
	Very delayed	6

TDM: therapeutic drug monitoring, IFIs: invasive fungal infections, HM: hematological malignancies, BAL: bronchoalveolar lavage.

**Table 3:** Diagnostic capacity for IFIs in 17 laboratories of participating Greek hospitals.

Question	Answer	%
Type of fungus diagnosed more frequently	*Candida* spp.	84
	*Aspergillus* spp.	16
Capacity for serology/molecular tests	Galactomannan	53
	β-d-glucan	13
	Mannan	20
	Anti-mannan	7
	PCR for fungi	7
Capacity for species identification	*Aspergillus*	10
	*Candida*	100
Capacity for susceptibly testing	Yes	88
	No	12
Capacity for TDM	Yes	6
	No	94

TDM: therapeutic drug monitoring, IFIs: invasive fungal infections.

**Table 4:** Treatment strategies for documented or presumed IFIs.

Question	Answer	%
Type IFIs treated during last year	Aspergillosis	32
	Invasive candidiasis	29
	Mucormycosis	22
	Fusariosis	14
	Other rare fungi	3
Use of antifungal prophylaxis	Yes	95
	No	5
Type of patients receiving antifungal prophylaxis	AML induction therapy	22
	AML consolidation therapy	18
	Lymphomas treated with purine analogues	11
	Allogeneic HSCT	11
	Lymphomas treated with monoclonal antibodies	8
	MM receiving chemotherapy	9
	MDS receiving chemotherapy	3
	All hematology patients treated in suboptimal hygiene conditions	9
	All hematology patients	2
Type of treatment strategy	Empirical	59
	Pre-emptive	21
	Targeted	20
Time to start empirical treatment in patients with fever and neutropenia	0–2 days	6
	3–5 days	52
	6–8 days	41
Factors affecting decisions for choosing specific antifungals for empirical treatment	Cost	28
	Availability of the drug in the pharmacy	26
	Guidelines of the hospital	25
	Underlying disease/chemotherapy	22
	International guidelines	20
	Antifungal spectrum of the drug	15
	Local epidemiology	11
	Registered indications of the drug	11
	Efficacy	11
	Safety	9

IFIs: invasive fungal infections, MM: multiple myeloma, AML: acute myeloid leukemia, MDS: myelodysplastic syndrome.
